# Mechanisms of Myocardial Ischemia in Hypertrophic Cardiomyopathy: Insights From Wave Intensity Analysis and Magnetic Resonance

**DOI:** 10.1016/j.jacc.2016.07.751

**Published:** 2016-10-11

**Authors:** Claire E. Raphael, Robert Cooper, Kim H. Parker, Julian Collinson, Vassilis Vassiliou, Dudley J. Pennell, Ranil de Silva, Li Yueh Hsu, Anders M. Greve, Sukh Nijjer, Chris Broyd, Aamir Ali, Jennifer Keegan, Darrel P. Francis, Justin E. Davies, Alun D. Hughes, Andrew Arai, Michael Frenneaux, Rod H. Stables, Carlo Di Mario, Sanjay K. Prasad

**Affiliations:** a NIHR Cardiovascular Biomedical Research Unit, Royal Brompton Hospital, London, United Kingdom; b Institute of Cardiovascular Medicine and Science, Liverpool Heart and Chest Hospital, Liverpool, United Kingdom; c Department of Bioengineering, Imperial College, London, United Kingdom; d National Institutes of Health, Bethesda, Maryland; e International Centre for Circulatory Health, Imperial College, London, United Kingdom; f University College London, London, United Kingdom; g Department of Cardiology, University of East Anglia, Norwich, United Kingdom

**Keywords:** angina, cardiovascular magnetic resonance, CMR, left ventricular outflow tract obstruction, perfusion, BCW, backward compression wave, BCW_tot_, backward compression wave, BEW, backward expansion wave, CFR, coronary flow reserve, FCW, forward compression wave, FCW_a_, additional forward compression wave, FEW, forward expansion wave, FEW_a_, additional forward expansion wave, HCM, hypertrophic cardiomyopathy, LGE, late gadolinium enhancement, LVOT, left ventricular outflow tract, MBF, myocardial blood flow, MPR, myocardial perfusion reserve, WIA, wave intensity analysis

## Abstract

**Background:**

Angina is common in hypertrophic cardiomyopathy (HCM) and is associated with abnormal myocardial perfusion. Wave intensity analysis improves the understanding of the mechanics of myocardial ischemia.

**Objectives:**

Wave intensity analysis was used to describe the mechanisms underlying perfusion abnormalities in patients with HCM.

**Methods:**

Simultaneous pressure and flow were measured in the proximal left anterior descending artery in 33 patients with HCM and 20 control patients at rest and during hyperemia, allowing calculation of wave intensity. Patients also underwent quantitative first-pass perfusion cardiac magnetic resonance to measure myocardial perfusion reserve.

**Results:**

Patients with HCM had a lower coronary flow reserve than control subjects (1.9 ± 0.8 vs. 2.7 ± 0.9; p = 0.01). Coronary hemodynamics in HCM were characterized by a very large backward compression wave during systole (38 ± 11% vs. 21 ± 6%; p < 0.001) and a proportionately smaller backward expansion wave (27% ± 8% vs. 33 ± 6%; p = 0.006) compared with control subjects. Patients with severe left ventricular outflow tract obstruction had a bisferiens pressure waveform resulting in an additional proximally originating deceleration wave during systole. The proportion of waves acting to accelerate coronary flow increased with hyperemia, and the magnitude of change was proportional to the myocardial perfusion reserve (rho = 0.53; p < 0.01).

**Conclusions:**

Coronary flow in patients with HCM is deranged. Distally, compressive deformation of intramyocardial blood vessels during systole results in an abnormally large backward compression wave, whereas proximally, severe left ventricular outflow tract obstruction is associated with an additional deceleration wave. Perfusion abnormalities in HCM are not simply a consequence of supply/demand mismatch or remodeling of the intramyocardial blood vessels; they represent a dynamic interaction with the mechanics of myocardial ischemia that may be amenable to treatment.

Hypertrophic cardiomyopathy (HCM) afflicts 1 in 500 of the general population (1). Chest pain affects up to one-half of patients and is believed to result from impaired myocardial perfusion (2). Although it is difficult to treat (3), severe perfusion abnormalities are independently predictive of death (4) and development of heart failure (5).

Several potential mechanisms have been proposed to explain these perfusion abnormalities, including increased oxygen requirements due to hypertrophy, impaired ventricular relaxation, anatomic abnormalities of intramyocardial arterioles, and left ventricular outflow tract (LVOT) obstruction 3, 6, 7, 8. However, the relative contribution of each factor, and the impact of LVOT obstruction compared with myocardial hypertrophy alone, remains unclear.

Wave intensity analysis (WIA) describes the waves that cause acceleration or deceleration of coronary blood (9), and it has allowed elucidation of the dominant mechanisms underlying coronary flow in structurally normal hearts (10) and aortic stenosis (11). Acceleration of coronary flow results from either compression waves originating from the aortic (proximal) end or expansion waves originating from the microcirculatory (distal) end. Separated wave intensity allows separation of the proximal and distal effects, whereas net wave intensity describes the net result of opposing waves during the cardiac cycle.

In the healthy heart, coronary flow is largely governed by 2 waves: the forward compression wave (FCW), which is generated by ventricular contraction and accelerates blood into the coronaries, and the backward expansion wave (BEW), which results from decompression of the microcirculation as the ventricle relaxes (Figure 1). The FEW decelerates coronary flow and occurs as ventricular contraction slows. Distally, the early and late backward compression waves (BCWs) occur during systole as the intramyocardial vessels are compressed (10).

**Figure 1:**
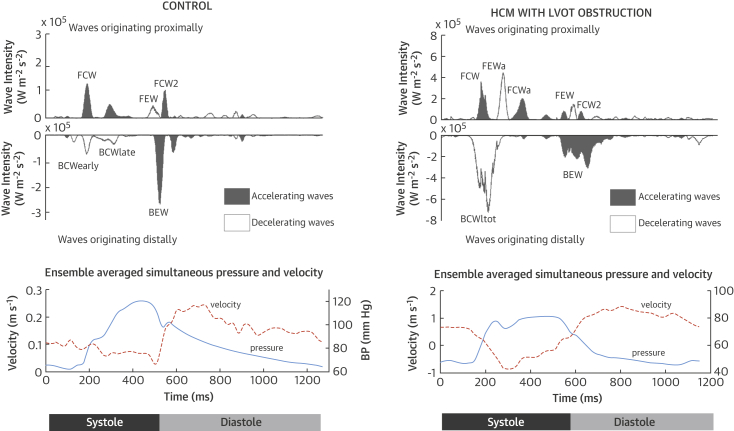
Separated Wave Intensity In the wave intensity analysis (WIA) pattern of patients at rest **(top)**, proximally originating waves are displayed above the axis and distally originating waves below the axis. Using a different scale, the ensemble averages **(bottom panel)** show the trajectory of pressure **(solid line)** and flow velocity **(dashed line)**. In control subjects, the dominant wave is the backward expansion wave (BEW), but in patients with hypertrophic cardiomyopathy (HCM), the backward compression wave (BCW) is dominant. Severe left ventricular outflow tract (LVOT) obstruction produces additional forward waves. BP = blood pressure; BCW_tot_ = backward compression wave total; FCW = forward compression wave; FCW_a_ = additional forward compression wave; FCW2 = forward compression wave 2; FEW = forward expansion wave; FEW_a_ = additional forward expansion wave.

In HCM, there are increased competing forces both proximally and distally. Proximally, there are changes in left ventricular (LV) contraction and relaxation and possible effects of LVOT obstruction; distally, there are changes due to compressed intramyocardial blood vessels and dysfunction of the microcirculation. Coronary flow abnormalities are common in HCM, with flow reversal during systole and higher flow velocities during diastole 12, 13, 14.

The goal of the present study was to elucidate the mechanism of myocardial ischemia and angina in HCM through the combination of coronary WIA and cardiac magnetic resonance (CMR) perfusion.

## Patients and Methods

Consecutive patients with HCM and an indication for coronary angiography were invited to participate. Control subjects had a structurally normal heart, atypical chest pain, an indication for coronary catheterization, and angiographically unobstructed coronary arteries. All participants provided written informed consent, and the study was approved by an independent ethics committee.

HCM was defined according to American Heart Association criteria (15). LVOT obstruction was defined at the time of cardiac catheterization as a resting gradient of ≥30 mm Hg. Exclusion criteria were as follows: 1) coronary artery disease (defined as >50% narrowing of epicardial coronary arteries >2 mm); 2) hypertension not controlled by medical therapy (>140/80 mm Hg at rest on 2 successive readings); and 3) valvular heart disease (excluding mitral regurgitation due to systolic anterior motion of the mitral valve).

Subjects were instructed to abstain from smoking and consumption of caffeine-containing substances and nitrate medications for 24 h before examination.

After pressure equalization with the pressure sensor at the tip of the guiding catheter, the Doppler velocity and pressure wire (ComboWire, Volcano Corporation, San Diego, California) was advanced in the proximal left anterior descending coronary artery with optimization of the Doppler signal. Simultaneous recordings of pressure, flow velocity, and electrocardiogram were obtained at a sampling rate of 200 Hz at rest and peak hyperemia after an intravenous infusion of adenosine (140 μg/kg/min) for 3 min. Data were similarly obtained in the proximal ascending thoracic aorta. LV pressure was recorded at rest and during hyperemia.

The duration of systole and diastole and time between onset of diastole (defined by using the dicrotic notch) (14) to peak coronary flow were calculated for each patient. The instantaneous coronary volume flow rate was calculated from the product of the instantaneous flow velocity and the coronary cross-sectional area measured by using quantitative coronary angiography (16). The mean volume coronary flow rate was averaged over the whole cardiac cycle. Coronary flow reserve (CFR) was calculated as the mean coronary volume flow rate throughout the cardiac cycle at maximal hyperemia divided by the mean coronary volume flow rate at rest. Coronary resistance was defined as pressure divided by flow. Mean coronary resistance was defined as the mean of the instantaneous resistance over the relevant period (systole, diastole, or the entire cardiac period).

### Wave intensity analysis

Data were analyzed by using a customized automated MATLAB program (MathWorks, Natick, Massachusetts) (10). Four to 5 beats of pressure and flow velocity data were ensemble-averaged, and a Savitzky-Golay filter (polynomial order 3, frame size 17) was used to calculate the time derivative of the data (17). The derivatives of pressure and flow at each time point were used to calculate the net wave intensity. Waves were categorized in terms of direction (forward [waves of proximal origin] or backward [waves of distal origin]) and associated pressure change (compression [a rise in pressure] or expansion [a fall in pressure]). The sum-of-squares method was used to determine the wave speed for calculation of the separated wave intensity (9). Because this method has been shown to be less accurate under conditions of low coronary resistance (18), only the net wave intensity was used to compare rest and hyperemia.

Separated wave intensity allows separation of the proximal and distal effects, whereas net wave intensity describes the net result of opposing waves during the cardiac cycle (Online Figure 1). For both separated wave intensity and net WIA, the cumulative wave intensity was defined as the integral of wave intensity over a given peak. For comparison between patients, each wave was also presented as a proportion of the total cumulative wave intensity (either separated or net), the percentage of separated cumulative wave intensity, and the percentage of separated net wave intensity, respectively (10). The proportion of total wave intensity comprising accelerating waves (forward compression and BEWs) was also calculated.

### CMR data acquisition and analysis

CMR data were collected on a 3-T MAGNETOM Skyra scanner (Siemens Corporation, Malvern, Pennsylvania). A balanced steady-state free precession sequence was used to obtain breath-hold cine images in 3 long-axis planes, followed by a contiguous short-axis stack through the ventricle. Myocardial first-pass perfusion imaging was performed by using a rate 3, parallel-accelerated balanced steady-state free precession sequence (19). Three short-axis images and an image of the arterial input function were acquired every cardiac cycle for 70 cycles. Initial proton density–weighted images were used for subsequent surface coil intensity correction.

CMR first-pass perfusion was recorded at hyperemia and rest. Adenosine was infused at 140 μg/kg/min by using the same protocol as in the invasive study. At peak hyperemia, a 0.05–mmol/kg bolus of gadolinium contrast (Gadovist, Bayer Schering Pharma AG, Berlin, Germany) was rapidly injected followed by a 25-ml saline flush. After acquisition of first-pass perfusion, adenosine was stopped, and a top-up 0.05-mmol/kg bolus of gadolinium was administered. Late gadolinium enhancement (LGE) imaging was performed by using an inversion recovery–prepared, segmented turbo fast low-angle shot sequence 10 min after gadolinium injection. The inversion recovery time was optimized to null normal myocardium. After 20 min, rest perfusion was similarly performed.

Ventricular volumes, function, and mass were assessed by using semi-automated CMRtools software (Cardiovascular Imaging Solutions, London, United Kingdom). Myocardial blood flow (MBF) was calculated by using customized software (Exelis Visual Information Solutions, Boulder, Colorado) to correct surface coil intensity bias and motion artifacts. MBF was quantified by using model-constrained deconvolution (20). LGE was quantified by using the full-width, half-maximum method, and findings are reported as a percentage of total LV mass using commercially available software (CMR42, Circle Cardiovascular Imaging, Calgary, Alberta, Canada).

### Statistical analysis

Statistical analysis was performed by using Stata version 12 (StataCorp LP, College Station, Texas). Normally distributed data are presented as mean ± SD. Comparisons between groups were performed by using the Student *t* test for 2 samples and analysis of variance for 3 variables; correlation was determined by using the Pearson correlation coefficient. Nonnormally distributed data are presented as median and interquartile range and compared by using the Mann-Whitney *U* test or Spearman’s rank test as appropriate. A p value <0.05 was considered significant.

## Results

Baseline characteristics are shown in Table 1. Of the 33 patients with HCM, 16 had resting LVOT obstruction (mean gradient 60 ± 36 mm Hg). Two patients with HCM had minor coronary irregularities, and the remainder had angiographically normal coronary arteries. All control subjects had angiographically normal coronary arteries. CFR was lower in patients with HCM compared with control subjects (1.9 ± 0.8 vs. 2.7 ± 0.9; p = 0.01) due to a higher resting mean coronary flow velocity; there was no significant difference in mean coronary flow velocity during hyperemia. Differences in cardiac cycle timings between patients with HCM and control subjects are shown in Table 2. Flow reversal during systole was seen in 10 (63%) of 16 patients with HCM with LVOT obstruction and 3 (18%) of 17 without LVOT obstruction. No control subjects had flow reversal.

**Table 1:** Patient Characteristics

	Control Group (n = 20)	HCM Group (n = 33)	p Value
Age, yrs	59 ± 15	54 ± 14	0.89
Male	12 (60)	24 (73)	0.34
Wall thickness, mm	10 ± 2	21 ± 4	<0.01
LVOT obstruction	0 (0)	16 (48)	<0.01
In NYHA functional class I/II/III	13/7/0	4/18/11	<0.01
NYHA functional class	1.6 ± 0.5	2.2 ± 0.6	0.03
History of ventricular tachycardia	0 (0)	3 (9)	0.17
Implantable cardioverter-defibrillator	0 (0)	2 (6)	0.26
Chest pain	16 (80)	24 (73)	0.55
Shortness of breath	4 (20)	26 (88)	<0.01
Syncope	0 (0)	9 (27)	0.01
LVEDVi, ml/m^2^	130 ± 42	71 ± 21	0.01
LVESVi, ml/m^2^	41 ± 24	18 ± 9	<0.01
LVEF, %	70 ± 8	73 ± 14	0.07
LVMi, g/m^2^	63 ± 15	115 ± 41	<0.01
RVEF, %	58 ± 5	65 ± 14	<0.01
LGE, % of total left ventricular mass	0 ± 0	22.3 ± 14.1	<0.01
Rest MBF, ml/kg/min	1.0 ± 0.3	1.1 ± 0.3	0.56
Hyperemic MBF, ml/kg/min	1.8 ± 0.5	1.5 ± 0.5	0.04
MPR	1.9 ± 0.5	1.4 ± 0.3	<0.01
Resting heart rate, beats/min	68 ± 22	64 ± 11	0.54
Resting systolic blood pressure, mm Hg	130 ± 16	116 ± 19	0.04
Resting diastolic blood pressure, mm Hg	70 ± 10	65 ± 15	0.26
LAD diameter, mm	3.1 ± 0.9	3.8 ± 0.6	0.03
LVEDP rest, mm Hg	14.2 ± 2.6	24.4 ± 8.3	<0.01
LVEDP hyperemia, mm Hg	13.6 ± 3.1	27.7 ± 7.1	<0.01
Comorbidities			
Hypertension	12 (60)	7 (21)	<0.04
Diabetes	2 (10)	1 (3)	0.29
Hypercholesterolemia	12 (60)	4 (12)	<0.01
Current smoker	4 (20)	4 (12)	0.44
Medications			
Beta-blockers	4 (20)	26 (79)	<0.01
ACE inhibitors	8 (40)	3 (9)	<0.07
Calcium-channel blockers	0 (0)	6 (18)	0.04

Values are mean ± SD, n (%), or n.

ACE = angiotensin-converting enzyme; HCM = hypertrophic cardiomyopathy; LAD = left anterior descending artery; LGE = late gadolinium enhancement; LVEDVi = left ventricular end-diastolic volume index; LVEDP = left ventricular end-diastolic pressure; LVEF = left ventricular ejection fraction; LVESVi = left ventricular end-systolic volume index; LVMi = left ventricular mass indexed to body surface area; LVOT = left ventricular outflow tract; MBF = myocardial blood flow; MPR = myocardial perfusion reserve; NYHA = New York Heart Association; RVEF = right ventricular ejection fraction.

**Table 2:** Coronary Flow and Cardiac Cycle Timing

	Rest			Hyperemia		
	Control Group	HCM Group	p Value	Control Group	HCM Group	p Value
Mean coronary flow velocity, m/s	0.19 ± 0.07	0.27 ± 0.11	0.001	0.49 ± 0.18	0.48 ± 0.18	0.78
Cycle length, ms	948 ± 267	937 ± 227	0.68	873 ± 148	864 ± 145	0.60
Duration of diastole, ms	527 ± 157	482 ± 82	0.21	304 ± 115	240 ± 120	0.02
Time between onset of diastole and peak coronary velocity, ms	109 ± 32	145 ± 72	0.007	114 ± 42	163 ± 76	0.009
Coronary resistance, mm Hg/ml/min	3.7 ± 1.3	1.9 ± 1.4	<0.001	1.3 ± 0.7	1.1 ± 1.4	0.46

Values are mean ± SD.

HCM = hypertrophic cardiomyopathy.

Fourteen (88%) patients with LVOT obstruction demonstrated a bisferiens pressure waveform in the proximal left anterior descending coronary artery, also observed in the proximal aorta. This finding was not seen in control subjects or in patients HCM without resting LVOT obstruction.

**Figure 2:**
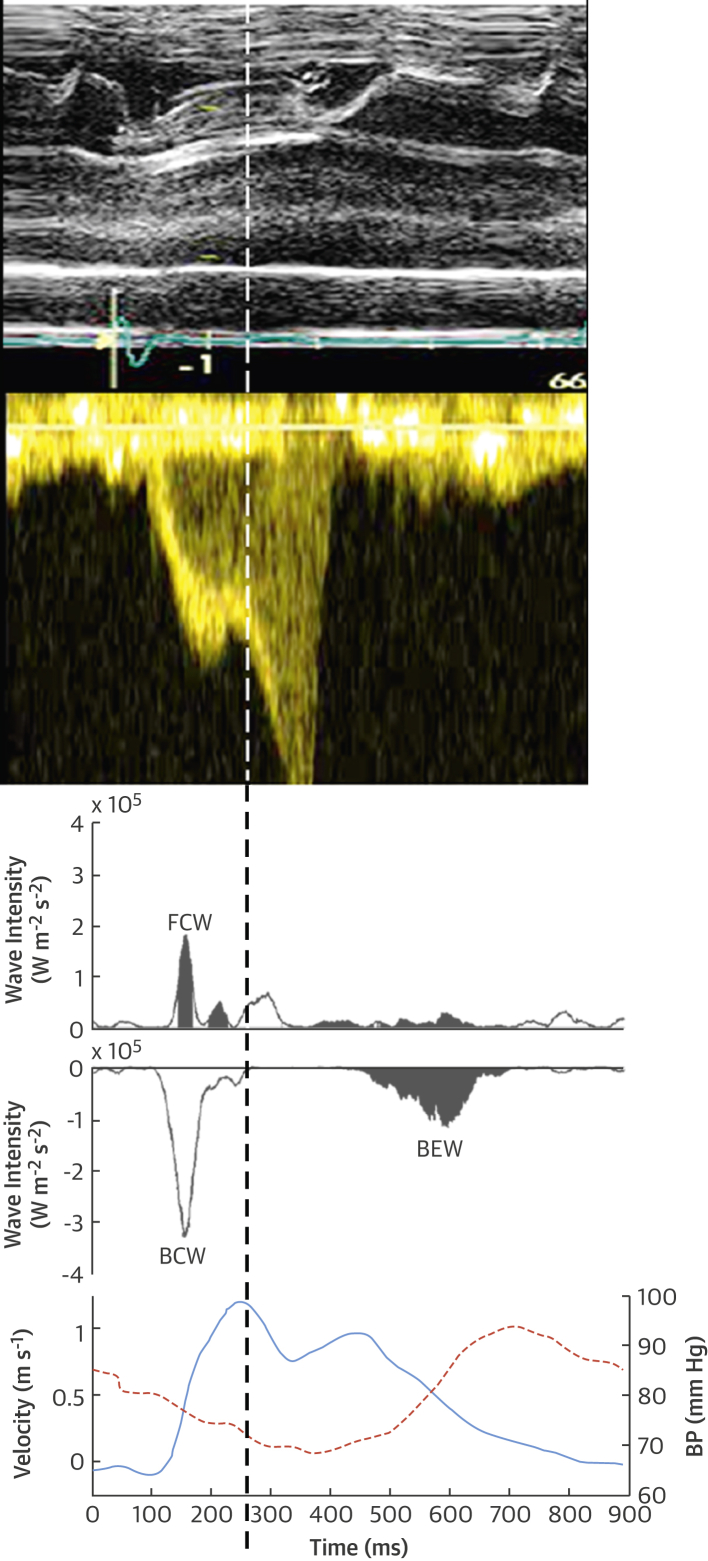
Origin of Additional Waves Resulting From LVOT Obstruction Obstruction of the LVOT due to systolic anterior motion of the mitral valve (**top panel**, M mode through mitral valve) results in a transient reduction in outflow (**middle panel**, continuous wave Doppler) and a decelerating wave (FEW_a_), which is transmitted into the coronary arteries. **(Bottom panel)** This action causes deceleration of coronary flow and a reduction in coronary pressure. The panels were aligned by the electrocardiogram R wave with a minimal time delay to account for distance between measurement sites. Abbreviations as in Figure 1.

Patients with HCM had a continuous backward compression wave (BCW_tot_) during early systole rather than the separate early and late backward compression waves seen in control subjects. In 14 of 16 patients with LVOT obstruction, there was an additional forward expansion wave (FEW_a_) during ventricular systole, and 9 of 16 patients had an additional forward compression wave (FCW_a_) after the FEW_a_ (Figure 1). These waves coincided with the transient drop and then rise in proximal left anterior descending coronary artery pressure seen in the bisferiens pressure waveform, coupled with a decrease in coronary flow velocity (Figure 2).

In HCM, a larger proportion of the total separated cumulative wave intensity comprised the BCW_tot_ and a smaller proportion by the BEW compared with control subjects (Table 3). There was no significant difference in any of the waves between the LVOT obstruction HCM group and the no-LVOT obstruction HCM group, with the exception of the additional FCW_a_ and FEW_a_, which were present only in the LVOT group. There was a significant correlation between the LVOT gradient and the FEW_a_ and FCW_a_ (rho = 0.82 [p < 0.001] and 0.40 [p = 0.011], respectively) but no correlation with the other waves.

**Table 3:** Separated Cumulative Wave Intensity

	Separated Cumulative Wave Intensity (W m^–2^s^–1^ × 10^6^)			Proportion of Separated Cumulative Wave Intensity (%)		
	Control Group	HCM Group	p Value	Control Group	HCM Group	p Value
FCW	6.6 ± 4.1	7.3 ± 5.1	0.61	26.8 ± 7.4	22.4 ± 10.9	0.09
FEW	2.8 ± 2.5	1.4 ± 1.2	0.005	9.9 ± 4.6	4.2 ± 2.6	<0.001
FEW_a_	0 ± 0	2.1 ± 2.6	<0.001	0 ± 0	4.1 ± 4.9	0.02
FCW_a_	0 ± 0	1.3 ± 1.9	0.002	0 ± 0	1.5 ± 4.1	0.08
FCW2	2.2 ± 1.8	1.3 ± 1.4	0.03	9.5 ± 4.1	2.3 ± 4.6	<0.001
BCW_tot_	4.9 ± 3.0	13 ± 8.3	<0.001	21.0 ± 6.2	38.2 ± 11.1	<0.001
BEW	8.3 ± 5.1	9.8 ± 6.5	0.4	32.8 ± 6.2	27.2 ± 7.9	0.006

Values are mean ± SD.

BCW_tot_ = backward compression wave total; BEW = backward expansion wave; FCW = forward compression wave; FCW2 = forward compression wave 2; FCW_a_ = additional forward compression wave; FEW = forward expansion wave; FEW_a_ = additional forward expansion wave; HCM = hypertrophic cardiomyopathy.

### Impact of hyperemia

**Figure 3:**
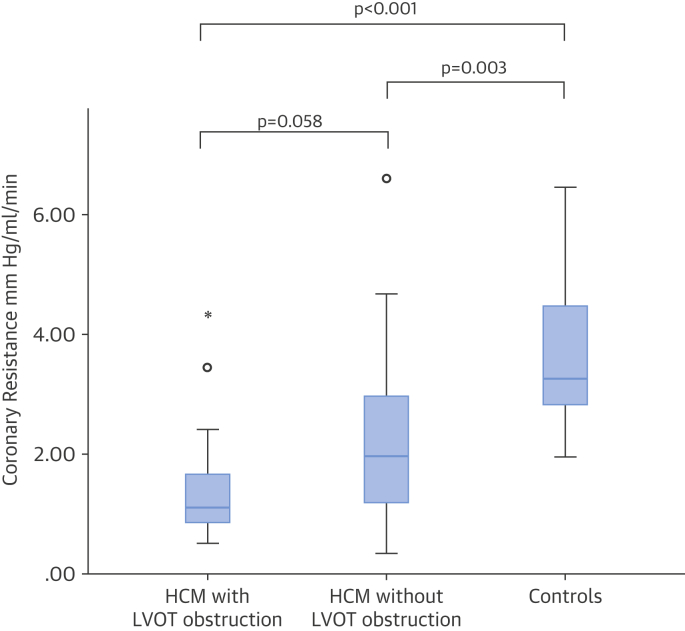
Resting Coronary Resistance Mean coronary resistance was defined as the mean of the instantaneous resistance over the entire cardiac period. Resistance was lowest in HCM patients with LVOT obstruction and highest in control subjects. Abbreviations as in Figure 1.

During hyperemia, coronary flow velocity increased and coronary resistance decreased in all patients. Patients with HCM had a lower resting mean coronary resistance than control subjects (Figure 3). Comparing patients with HCM versus control subjects, resting MBF was similar, but hyperemic MBF and myocardial perfusion reserve (MPR) were lower (Table 1).

At rest, there was a higher BCW_tot_ and lower FEW in patients with HCM compared with control subjects. The FCW was significantly lower, but there was no significant difference in the BEW. During hyperemia, there was a significant increase in the size of all waves in both groups. The FCW was smaller and the BCW_tot_ was larger in patients with HCM compared with control subjects (Table 4; absolute values, Online Table 1). There was no difference in the BEW. The ratio of accelerating/decelerating waves at rest was 3.3 ± 1.4 in the control group and 1.9 ± 2.0 in the HCM group (p < 0.01). During hyperemia, the ratio was 2.2 ± 1.3 in the control group and 1.3 ± 1.1 in the HCM group (p < 0.01).

**Table 4:** Proportion of Net Cumulative Wave Intensity (%) at Rest and Hyperemia

	Rest			Hyperemia		
	Control Group	HCM Group	p Value	Control Group	HCM Group	p Value
FCW, %	30.1 ± 14.9	15.7 ± 17.5	0.004	24.7 ± 15.1	11.7 ± 13.1	0.002
FEW, %	8.8 ± 7.3	2.5 ± 3.1	<0.001	6.9 ± 6.9	2.7 ± 3.4	0.006
FCW_a_, %	0 ± 0	7.4 ± 7.2∗	<0.001	0 ± 0	9.4 ± 9.4∗	<0.001
FEW_a_, %	0 ± 0	3.9 ± 2.7∗	<0.001	0 ± 0	2.2 ± 1.3∗	<0.001
FCW2, %	6.0 ± 6.7	3.2 ± 8.1	0.21	2.1 ± 3.9	1.0 ± 2.1	0.17
BCW_tot_, %	16.6 ± 9.5	38.3 ± 19.2	<0.001	27.9 ± 12.6	43.2 ± 14.2	<0.001
BEW, %	38.6 ± 13.5	37.2 ± 12.3	0.70	38.4 ± 10.3	38.7 ± 11.2	0.90

Values are mean ± SD.

Abbreviations as in Tables 1 and 3.

∗ Means were calculated only for the patients with the additional wave (FCW_a_, n = 14; FEW_a_, n = 9).

**Figure 4:**
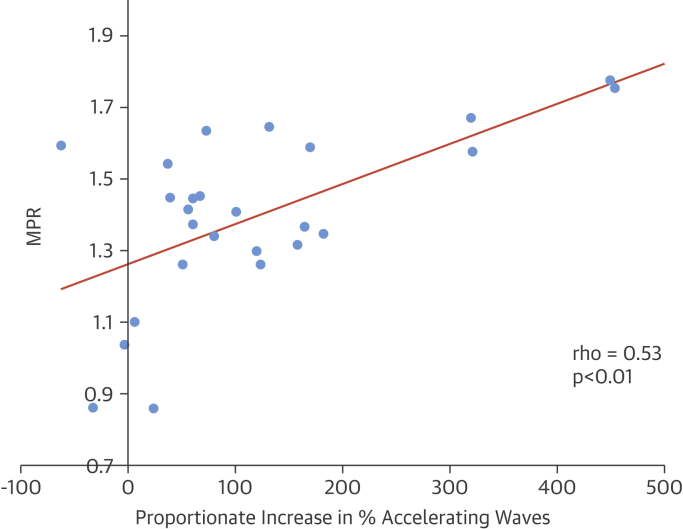
Coronary Wave Intensity and Myocardial Perfusion in HCM The myocardial perfusion reserve (MPR) was measured using cardiac magnetic resonance imaging and compared with the changes in wave intensity between rest and hyperemia. There was a significant correlation between the MPR and the proportionate increase in percentage of accelerating waves during hyperemia. HCM = hypertrophic cardiomyopathy.

MPR correlated with the percent increase in the proportion of accelerating waves (rho = 0.53; p < 0.01) (Figure 4). There was no significant correlation between the percent BCW_tot_ at rest and the MBF at rest (r = –0.25; p = 0.14), but a significant correlation was noted between the proportion of accelerating waves at rest and MBF at rest (r = 0.35; p = 0.02). There was no relationship between MPR and wall thickness (r = 0.3; p = 0.12), LV mass (r = –0.17; p = 0.39), or percent LGE (–0.012; p = 0.95). There was no relationship between percent LGE and wave intensity measures either at rest or hyperemia.

## Discussion

Microvascular ischemia in HCM is associated with chest pain, clinical deterioration, diastolic dysfunction, and adverse prognosis 3, 4, 5, 8. A pressing and unmet need in HCM is a better understanding of the mechanisms driving myocardial ischemia (15). Multiple factors contribute, including the following: increased wall thickness results in both a supply/demand mismatch and increased compression of the microcirculation during systole; LVOT obstruction results in high intracavity pressures during ventricular systole; and the microcirculatory vessels themselves have been shown to be abnormal on histologic analysis (21). Most pharmacotherapies in HCM have nonspecific actions, and there is consequently still a high burden of morbidity (22).

This study is the first to use wave intensity to provide detailed analysis of factors affecting coronary flow in HCM and to measure the impact on myocardial perfusion. Importantly, we assessed the interplay of upstream and downstream determinants of microvascular ischemia and described the dominant mechanisms.

### Mechanisms of impaired myocardial perfusion

Perfusion abnormalities in HCM have often been assumed to result simply from supply/demand mismatch; however, we recorded no correlation between wall thickness or LV mass and MPR. Rather, we showed that perfusion abnormalities result from dynamic changes in myocardial mechanics throughout the cardiac cycle.

Three mechanisms were highlighted that result in impaired myocardial perfusion in HCM and which might result in symptoms of chest pain and shortness of breath (Central Illustration). All of these mechanisms might occur separately or simultaneously in a patient. First, extravascular compressive deformation of the intramyocardial precapillary arteries and arterioles during ventricular systole produces a large BCW. This decelerating wave accounts for the retrograde coronary flow in systole seen in some individuals with HCM. Second, impaired ventricular relaxation results in a proportionately smaller BEW. Finally, in some patients with HCM, transient LVOT obstruction decreases the proximal driving pressure, resulting in an additional forward deceleration wave that is transmitted into the proximal coronary artery and diminishes coronary forward flow. Coronary flow in HCM is therefore deranged, with opposing forces acting throughout the cardiac cycle. Myocardial perfusion is determined by the relative proportion of accelerating and decelerating waves.

**Central Illustration:**
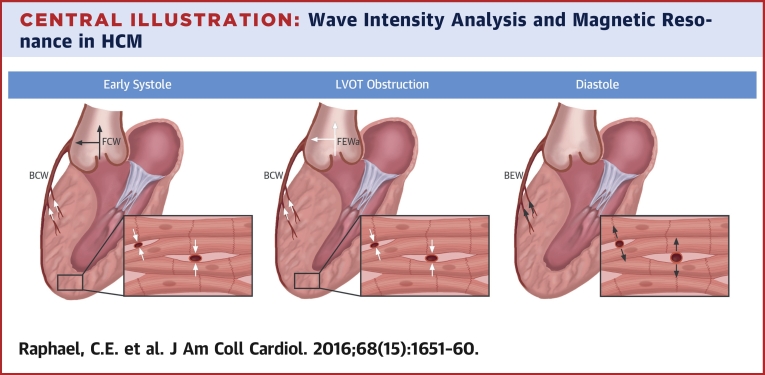
Wave Intensity Analysis and Magnetic Resonance in HCM There are 3 main mechanisms that result in abnormal myocardial perfusion in hypertrophic cardiomyopathy (HCM) involving waves causing coronary flow acceleration **(black arrows)** and those leading to deceleration (**white arrows**; each **arrow** is in the direction of the underlying wave, rather than the direction of coronary flow). Compression and deformation of the microcirculation result in a large backward compression wave (BCW) during ventricular systole. In patients with severe left ventricular outflow tract (LVOT) obstruction, an additional forward deceleration wave decreases the proximal driving pressure during mid-to-late systole, further diminishing coronary forward flow. During diastole, impaired ventricular relaxation results in a proportionately smaller backward expansion wave (BEW). FCW = forward compression wave; FEW_a_ = additional forward expansion wave.

Compression and deformation of the pre-capillary arteries and arterioles during ventricular systole result in a very large BCW, sufficient to stop or even reverse flow in the proximal coronary arteries. Blood is squeezed back from the intramyocardial vessels toward the epicardial coronary arteries, and the increased compression of the arterioles results in higher impedances within these vessels.

The lower coronary resistance in HCM during diastole resulted in higher diastolic coronary flow in the present study patients. This finding suggests that despite the adverse remodeling and reduction in vessel density observed in HCM (21), vasodilation of the microcirculation is sufficient to normalize the myocardial perfusion at rest, at the expense of the CFR.

In control subjects, the ratio of the net cumulative wave intensity of the FCW to the BCW was approximately 2:1 (Table 4), resulting in acceleration of coronary flow during early systole. In HCM, this ratio was reversed to approximately 1:2, causing deceleration during early systole. The situation was worsened during hyperemia, in which the ratio was reduced to 1:4, and reversal of blood flow direction was even greater.

As the ventricle relaxes, compression of the intramyocardial vessels is relieved, generating a BEW that accelerates coronary flow. In the present HCM cohort, the BCW during ventricular contraction was greater than the BEW during ventricular relaxation, which was the opposite of the pattern seen in control subjects. Patients with HCM typically have diastolic impairment 23, 24 with a prolonged time to reach peak diastolic flow compared with control subjects and a raised end-diastolic pressure. Diastolic dysfunction results from increased wall thickness and myocardial fibrosis, leading to delayed relaxation and increased passive stiffness. It is therefore unsurprising that BEW did not correlate with the percentage of replacement fibrosis alone.

### Impact of LVOT obstruction

Patients with severe LVOT obstruction exhibited a bisferiens pressure wave in the proximal aorta. This outcome has previously been shown to result from transient obstruction of the LVOT (25). The bisferiens pressure wave is transmitted into the carotid artery, where it can be palpated clinically (26). We have shown that it is also transmitted into the proximal coronary artery in the form of FEW_a_, followed by FCW_a_ when the obstruction is relieved.

Although a larger BCW or smaller BEW might be expected in patients with LVOT obstruction compared with those without, clinically HCM is extremely heterogeneous. Both waves will be affected by the degree of diastolic dysfunction, myocardial fibrosis, and ventricular contractility; it is therefore not surprising that division of the present patient cohort according to presence of LVOT obstruction yielded no significant differences between the 2 subgroups.

The relative timing of systole and diastole further disadvantages patients with LVOT obstruction, which prolongs the duration of LV ejection at the expense of the diastolic phase (27). Despite the favorable reduction in microcirculatory resistance during diastole, the duration of diastolic coronary flow is reduced. The situation is exacerbated at higher heart rates, with an even shorter diastolic time despite increased basal metabolic requirements.

### Wave intensity during hyperemia

Resting coronary flow was higher in patients with HCM compared with control subjects, although myocardial perfusion was similar. Patients with HCM had both a reduced CFR and MPR. The ratio of accelerating/decelerating waves was lower in patients with HCM compared with control subjects, both at rest and during hyperemia. Myocardial perfusion is complex and mediated by waves generated both proximally and distally throughout the cardiac cycle. Adenosine produces a reduction in microcirculatory resistance due to dilation of small blood vessels and therefore an increase in intramyocardial blood volume. The subsequent BCW generated by compression of the intramyocardial blood vessels has a proportionately greater increase with adenosine compared with the other waves.

### Impact on potential treatment strategies

WIA might explain the improvement in myocardial perfusion seen with beta-blockers and calcium channel blockers (28). Both increase diastolic duration and reduce myocardial contractility, theoretically leading to a reduction in BCW. Improvements in myocardial perfusion 29, 30 and CFR (31) after septal reduction therapy have been assumed to result from reduction in intracavity pressures and reduced microvascular compression. Benefit might also result from elimination of the FEW_a_.

Treatment strategies that reduce decelerating waves, either of proximal or distal origin, will improve the efficiency of coronary flow and should therefore improve myocardial perfusion. Because perfusion abnormalities are common in HCM, treatments that prolong diastolic duration may be beneficial in all patients, not just those who are symptomatic.

### Study limitations

Recruited patients had a clinical indication for coronary angiography. Patients with HCM were therefore symptomatic, and their results may not be generalizable to the asymptomatic population. However, the patients recruited were representative of the subset of HCM that poses the greatest management challenge for clinicians. Control subjects typically had risk factors for coronary disease, including hypertension and diabetes, and had low-to-normal values of MPR; therefore, a degree of mild microvascular disease may have been present. If completely healthy control subjects had been recruited, there may have been even greater differences compared with those with HCM. Use of beta-blockers and angiotensin-converting enzyme inhibitors differed between the 2 populations, and we did not adjust for this factor in the analysis.

The mean age of patients with HCM was 54 years (range 22 to 74 years). Accordingly, our findings cannot be generalized to the pediatric or adolescent population. However, the results seemed similar in the 6 patients <40 years of age compared with the rest of the sample.

Separated WIA requires knowledge of the local wave speed, and the use of the sum-of-squares estimate for wave speed introduces some uncertainty into the calculations. However, small errors in the wave speed have been shown to have little effect on the pattern of separated wave intensity (32); moreover, the net WIA, which does not depend on estimation of wave speed, produced similar results.

## Conclusions

WIA in patients with HCM demonstrated that coronary flow abnormalities occur due to opposing proximal and distal effects resulting from compression and deformation of the intramural blood vessels and impaired ventricular relaxation. LVOT obstruction might generate an additional deceleration wave. These abnormalities result in impaired myocardial perfusion.

Perspectives **COMPETENCY IN MEDICAL KNOWLEDGE:** Myocardial perfusion abnormalities in patients with HCM are caused by compression of the microcirculation, LVOT obstruction, and impaired ventricular relaxation rather than a supply/demand mismatch or microcirculatory remodeling.

**TRANSLATIONAL OUTLOOK:** Future research should assess whether treatment of perfusion abnormalities can improve clinical outcomes in patients with HCM.
