# Supplementary material for: Mechanisms of Myocardial Ischemia in Hypertrophic Cardiomyopathy: Insights From Wave Intensity Analysis and Magnetic Resonance
Source: J Am Coll Cardiol. 2016 Oct 11;68(15):1651–60. doi: 10.1016/j.jacc.2016.07.751 (PMC5054113; doi:10.1016/j.jacc.2016.07.751)
Supplement: Online Figure 1 and Online Table 1 [file mmc1.docx]

**Online Figure 1. An example trace of separated and net wave intensity analysis in a patient with HCM and LVOT obstruction** Separated wave intensity allows separation of the proximal and distal effects while net wave intensity describes the net result of opposing waves during the cardiac cycle. In HCM, separated wave intensity analysis allows examination of the different mechanisms acting proximally and distally: proximally the FCW accelerates blood while distally, the BCW decelerates blood. Since the BCW is very large in this example, the net wave intensity is in the backward direction. The FEWa and FCWa occur at a point where there are minimal effects distally and so they are similar in both the separated and net wave intensity schematics. During diastole, the BEW is much larger than the FEW or the FCW2 and therefore the net wave intensity is all distal.


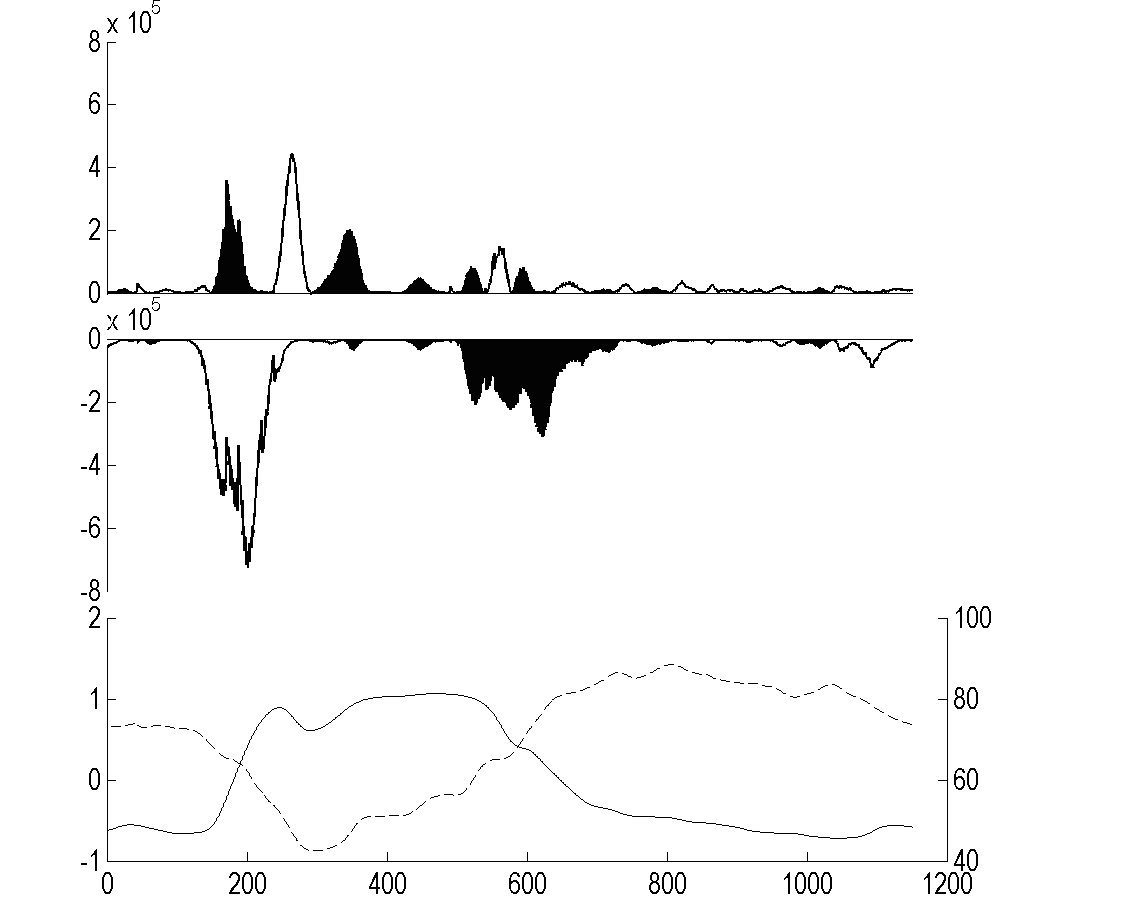

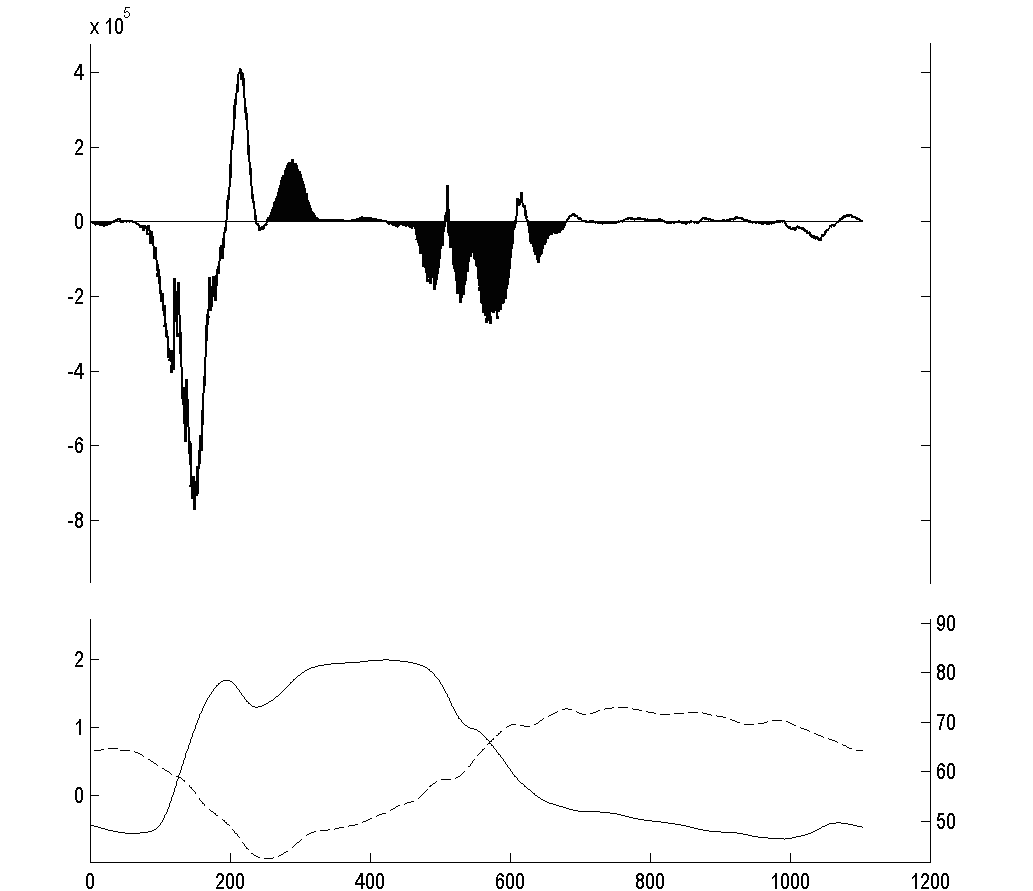


**Online Table 1**: Net Wave Intensity at rest and during hyperemia

|  | Rest/Wm^-2^s^-1^ x 10^6^ | | |  | Hyperemia/Wm^-2^s^-1^ x 10^6^ | | |
| --- | --- | --- | --- | --- | --- | --- | --- |
|  | **Control** | **HCM** | **p** |  | **Control** | **HCM** | **p** |
| Forward compression wave (FCW) | 4.1±3.3 | 2.5±3.5 | 0.11 |  | 6.6±5.3 | 3.9±4.8 | 0.07 |
| Forward expansion wave (FEW) | 1.5±1.9 | 0.3±0.4 | 0.01 |  | 1.8±2.5 | 0.8±1.3 | 0.11 |
| Forward expansion wave additional (FEWa) | 0±0 | 0.4±1.0 | 0.48 |  | 0±0 | 4.9±4.9 | <0.01 |
| Forward compression wave additional (FCWa) | 0±0 | 0.2.±0.7 | <0.01 |  | 0±0 | 1.8±1.4 | 0.01 |
| 2nd forward compression wave (FCW2) | 0.6±0.7 | 1.3±1.3 | <0.01 |  | 0.2±3.4 | 1.8±2.5 | 0.34 |
| Backward compression wave (BCWtot) | 2.0±1.7 | 7.5±7.0 | <0.01 |  | 12.6±11.9 | 16.1±13.5 | <0.01 |
| Backward expansion wave (BEW) | 5.2±3.7 | 6.8±5.5 | 0.21 |  | 9.2±5.3 | 14.4±12.1 | 0.04 |
| Accelerating waves | 9.9±5.5 | 10.2±7.2 | 0.83 |  | 16.0±9.4 | 19.2±15.1 | 0.35 |
| Decelerating waves | 3.5±2.8 | 8.4±7.7 | <0.01 |  | 8.4±5.1 | 19.0±17.0 | <0.01 |
